# Post-Drilling Changes in Seabed Landscape and Megabenthos in a Deep-Sea Hydrothermal System, the Iheya North Field, Okinawa Trough

**DOI:** 10.1371/journal.pone.0123095

**Published:** 2015-04-22

**Authors:** Ryota Nakajima, Hiroyuki Yamamoto, Shinsuke Kawagucci, Yutaro Takaya, Tatsuo Nozaki, Chong Chen, Katsunori Fujikura, Tetsuya Miwa, Ken Takai

**Affiliations:** 1 Department of Marine Biodiversity Research, Japan Agency for Marine-Earth Science and Technology (JAMSTEC), 2–15 Natsushima, Yokosuka, Kanagawa, 237–0061, Japan; 2 Research and Development Center for Submarine Resources, Japan Agency for Marine-Earth Science and Technology (JAMSTEC), 2–15 Natsushima, Yokosuka, Kanagawa, 237–0061, Japan; 3 Laboratory of Ocean-Earth Life Evolution Research (OELE), Japan Agency for Marine-Earth Science and Technology (JAMSTEC), 2–15 Natsushima, Yokosuka, Kanagawa, 237–0061, Japan; 4 Department of Subsurface Geobiological Analysis and Research (D-SUGAR), Japan Agency for Marine-Earth Science and Technology (JAMSTEC), 2–15 Natsushima, Yokosuka, Kanagawa, 237–0061, Japan; 5 Department of Systems Innovation, School of Engineering, The University of Tokyo, 7-3-1 Hongo, Bunkyo-ku, Tokyo, 113–8656, Japan; 6 Department of Zoology, University of Oxford, South Parks Road, Oxford, OX1 3PS, United Kingdom; 7 Marine Technology and Engineering Center (MARITEC), Japan Agency for Marine-Earth Science and Technology (JAMSTEC), 2–15 Natsushima, Yokosuka, Kanagawa, 237–0061, Japan; Universite Pierre et Marie Curie, FRANCE

## Abstract

There has been an increasing interest in seafloor exploitation such as mineral mining in deep-sea hydrothermal fields, but the environmental impact of anthropogenic disturbance to the seafloor is poorly known. In this study, the effect of such anthropogenic disturbance by scientific drilling operations (IODP Expedition 331) on seabed landscape and megafaunal habitation was surveyed for over 3 years using remotely operated vehicle video observation in a deep-sea hydrothermal field, the Iheya North field, in the Okinawa Trough. We focused on observations from a particular drilling site (Site C0014) where the most dynamic change of landscape and megafaunal habitation was observed among the drilling sites of IODP Exp. 331. No visible hydrothermal fluid discharge had been observed at the sedimentary seafloor at Site C0014, where *Calyptogena* clam colonies were known for more than 10 years, before the drilling event. After drilling commenced, the original *Calyptogena* colonies were completely buried by the drilling deposits. Several months after the drilling, diffusing high-temperature hydrothermal fluid began to discharge from the sedimentary subseafloor in the area of over 20 m from the drill holes, ‘artificially’ creating a new hydrothermal vent habitat. Widespread microbial mats developed on the seafloor with the diffusing hydrothermal fluids and the galatheid crab *Shinkaia crosnieri* endemic to vents dominated the new vent community. The previously soft, sedimentary seafloor was hardened probably due to barite/gypsum mineralization or silicification, becoming rough and undulated with many fissures after the drilling operation. Although the effects of the drilling operation on seabed landscape and megafaunal composition are probably confined to an area of maximally 30 m from the drill holes, the newly established hydrothermal vent ecosystem has already lasted 2 years and is like to continue to exist until the fluid discharge ceases and thus the ecosystem in the area has been altered for long-term.

## Introduction

In the last few decades, deep-sea hydrothermal ecosystems have been under increasing threat from various anthropogenic activities either underway or planned [1–3]. Recent technological developments have overcome the barrier of water depth and distance from shore, allowing the exploitation of previously inaccessible areas [4]. This has boosted the continuous expansion of anthropogenic activities in the hydrothermal vent ecosystems, including the exploitation of valuable mineral resources [5]. Hydrothermal vent sites produce seafloor massive sulfide (SMS) deposits with high-grade ores, giving them an attractive commercial prospect for mining [1,2].

Many potential impacts on the benthic community from mining activities are predicted: habitat loss and degradation, modification of fluid flux regimes, changes in diversity, and change of habitat conditions [1,3,6]. Although there is no case of commercial based seafloor mining on hydrothermal vent area so far, we can estimate the impacts or effects from mining activities from case studies of natural and artificial disturbances, such as volcanism and drilling [7]. Although disturbance caused by drilling may be different from proposed mining methods, including mechanical cutting, grabbing and dredging of vent chimney and hydrothermal deposits, impacts from drilling have the potential to serve as supporting evidence when assessing the risk of mining operation.

At present, the impact of drilling at hydrothermal systems is poorly understood (e.g. [8]). Most studies on the environmental impacts associated with deep-sea exploration (or commercial) drilling have been conducted in oil and gas field (e.g. [9–13]), focusing on how drilling deposits (cuttings and mud) affect mortality and survival rate of benthic animals (e.g. [12,11,10,14]). The drilling impact on seabed landscape and associated megabenthos in hydrothermal fields is likely to differ from these cases. Potential impacts that may be expected include the discharge of drilling deposits on to the seabed and subsequently high-temperature hydrothermal fluids from subseafloor. It is conceivable that the fluid discharges will attract recruits from the surrounding vent communities, creating ‘artificial’ hydrothermal vent ecosystems [7]. Understanding how the benthic community responds to drill-induced disturbance will help shape future ecosystem conservation strategy in anticipation of upcoming SMS mining activities. It is therefore crucial to conduct monitoring and assessment of the effect of drilling on the benthic community of deep-sea hydrothermal fields.

In September 2010, Integrated Ocean Drilling Program (IODP) Expedition 331 was carried out at the Iheya North hydrothermal field in the Okinawa Trough, Japan using the deep-sea drilling vessel *Chikyu* to investigate active subseafloor microbial communities associated with the physical and chemical variation of hydrothermal fluid flow [15,16]. The expedition established several drilling-induced, ‘artificial’ hydrothermal vents [16,17]. These artificial vents provided unique research opportunities for estimating the influence of anthropogenic drilling to the benthic communities of deep-sea hydrothermal ecosystems. As a part of an environmental impact assessment, we surveyed the changes in the seabed landscape and habitat as well as the abundance and composition of the megafaunal benthic community around the drill holes for over 3 years. Our focuses in this study are to examine the extent and persistence of the effects of the drilling operations in the area and to elucidate how the habitat condition as well as the benthic megabenthos communities are altered by the drilling.

## Materials and Methods

### Ethics statement

The location for this study was not privately owned or protected in any way and no specific permits were required for the described field studies and sample collection. The field studies did not involve any endangered or protected species. No invertebrate megafaunal specimens were collected in this work, as it was carried out using video techniques.

### Study location

**Fig 1:**
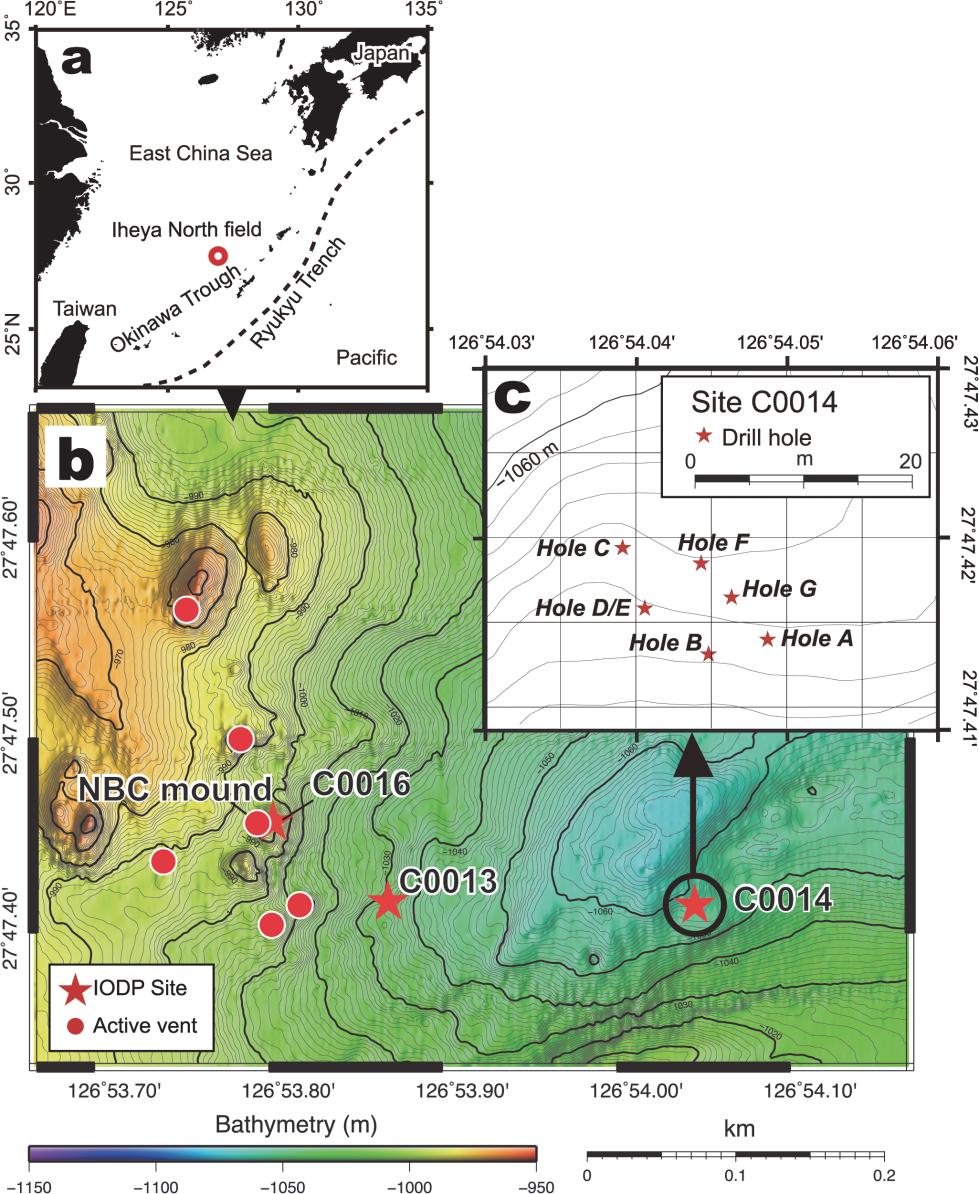
Maps of the study area. (a) Location of the Iheya North hydrothermal field, Okinawa Trough. (b) An event map of the Iheya North field. Red stars and circles represent the IODP drilling sites (C0013, C0014 and C0016) and hydrothermal fluid venting sites, respectively. (c) Location of drill holes at Site C0014 (Holes A-G).

Situated approximately 150 km off the Okinawa Island, Japan, the Iheya North field (27°45’-50’N; 126°53’-55’E) in the Okinawa Trough is a deep-sea hydrothermal field with a depth of ca. 1,000 m. Two decades of investigation since its discovery has revealed its geological background, fluid chemistry and microbiological characteristics [15,17–20]. Among the many active chimney sites, the 30 m high North Big Chimney (NBC) mound is the activity center of the field (Fig 1B, [18]). The Iheya North field is characterized by thick soft sediments offering habitats for endobenthic invertebrates even around hydrothermal vent site, allowing both vent-type and seep-type communities to exist in the area [21,22]. Representative species of megabenthic vent animals in the Iheya North field are *Shinkaia crosnieri* galatheid crab, *Alvinocaris longirostris* shrimp, *Paralvinella* polychaetes, *Paralomis* lithodid crab, and *Bathymodiolus japonicus* and *B*. *platifrons* mussels. Representative species of the geofluid seepage zone of the Iheya North vent field is the endobenthic deep-sea clam, *Calyptogena okutanii*, which colonizes the sedimentary seafloor [23].

**Fig 2:**
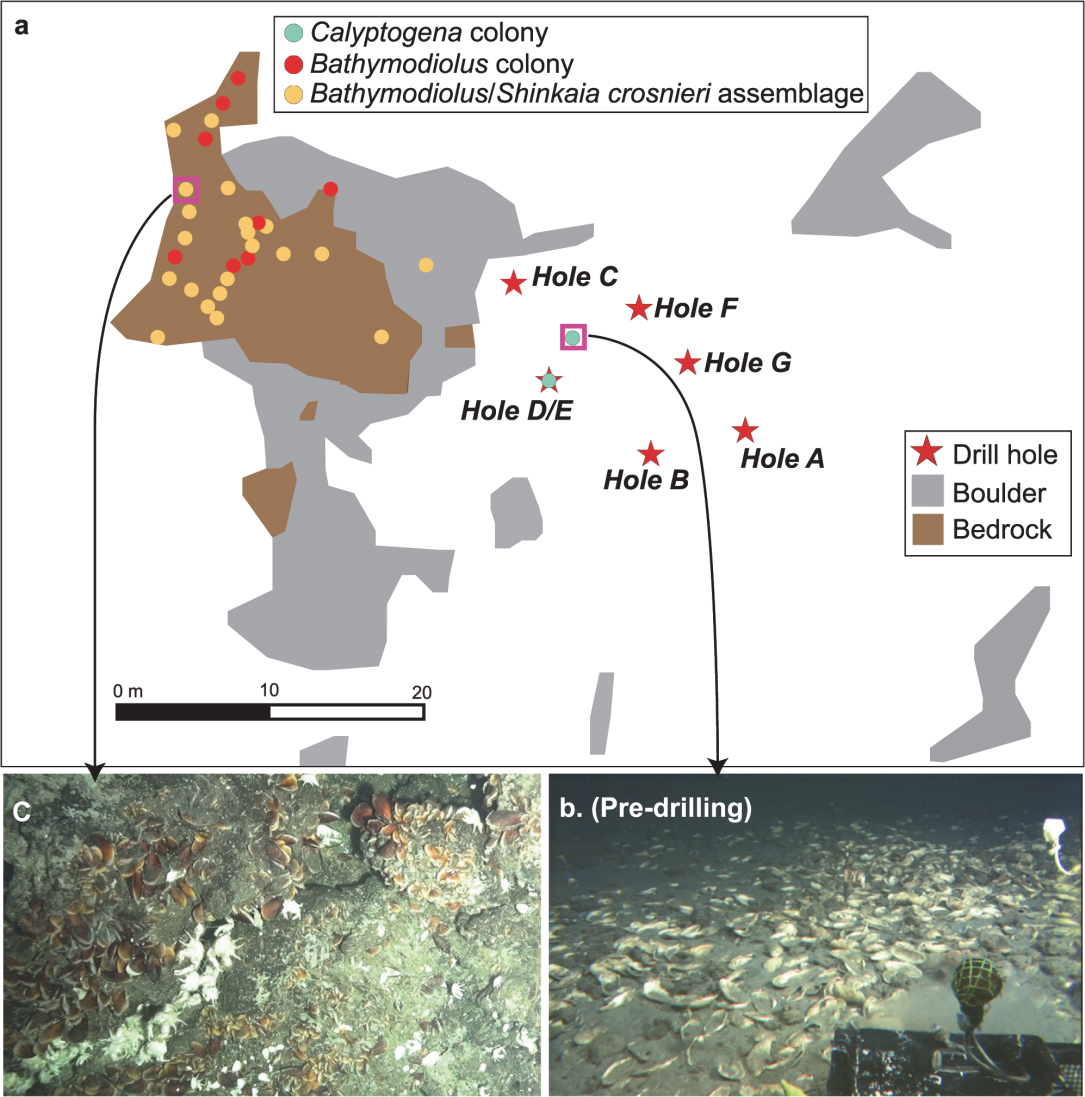
Habitat map around Site C0014. (a) Drill holes and colonies of *Calyptogena okutanii* clams and *Bathymodiolus* mussels and assemblages of *Bathymodiolus* mussels and *Shinkaia crosnieri* galatheid crabs. (b) Pre-drilling seabed landscape near Hole D/E with *C*. *okutanii* clam colonies. (c) An example of *Bathymodiolus* mussels and *S*. *crosnieri* galatheid crabs assemblage.

Of the several sites drilled during the IODP Expedition 331 (Fig 1B, see also [16]), Site C0014 is located 450 m east of the NBC mound. Around Site C0014, both active and non-active chimneys as well as apparent hydrothermal fluid discharges were not identified through visual observations by previous submersible surveys [21]. This site was characterized by several *C*. *okutanii* clam colonies, which had been identified for more than 10 years before the drilling event, indicating the colonies were likely supported by seepage of hydrothermal fluid input [16,17]. In total, seven holes were drilled at Site C0014 (i.e., Holes A-G) in a narrow area within 10 m radius, overlapping with the *Calyptogena* colonies (see red stars in Figs 1C and 2A). The holes were located at 1,060 m depth (Table 1). Hole G penetrated the deepest (136.7 m below the seafloor, mbsf), and the penetration depths of the other holes (Holes A-F) ranged from 4.2 to 44.5 mbsf (Table 1). Holes D and E were very closely located and they became one hole after the wall between them broke down (Hole D/E hereafter). At 11 months after the drilling operation only Holes D/E, B and G were visibly recognizable while the other holes had collapsed and filled up due to their shallow penetration (~6.5 mbsf).

**Table 1:** Drill hole summary at Site C0014 in the Iheya North hydrothermal field, Okinawa Trough.

Hole	Latitude (N)	Longitude (E)	Water depth	Hole depth
			(m)	(mbsf)
A	27°47.4140'	126°54.0487'	1059.5	6.5
B	27°47.4131'	126°54.0448'	1059.0	44.5
C	27°47.4194'	126°54.0391'	1060.0	6.5
D	27°47.4158'	126°54.0406'	1060.0	16.0
E	27°47.4158'	126°54.0406'	1060.0	35.0
F	27°47.4185'	126°54.0443'	1060.8	4.2
G	27°47.4165'	126°54.0463'	1059.8	136.7

**Fig 3:**
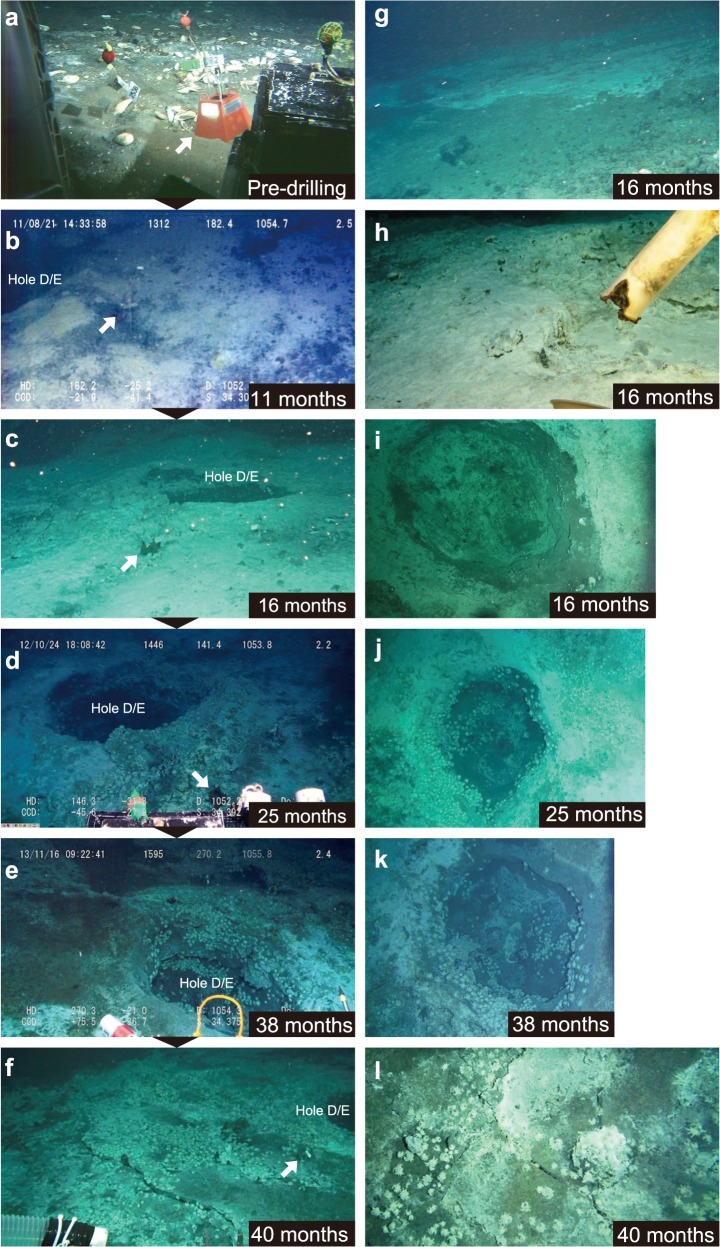
Temporal sequence of landscape at/around Hole D/E. Arrows in a-f indicate the sedimentation chamber base, which was placed pre-drilling (a). Photo g shows the edge of cuttings and photo h shows the bottom of the sedimentation chamber at 16 months post-drilling. Photos i-k are perpendicular images of Hole D/E at 16, 25 and 38 months post-drilling, respectively. Photo l is a perpendicular image of the bottom substratum at 40 months post-drilling, near Hole D/E.

The multiple drilling operations penetrated the repeated hard layers that might have served as cap rocks, and high-temperature hydrothermal fluid were discharged from the holes as well as in the shallower sediments surrounding the holes [15–17]. A casing pipe was deployed down to ca. 120 mbsf at Hole G fixed with a corrosion cap (open outlet pipe) mounted on the gimballed guide base [15,16]. After casing and capping, diffusing hydrothermal fluid discharged not from the corrosion cap outlet but from the seafloor through the annulus, the space between the wall of the hole and the casing pipe at Hole G; the temperature of the diffusing fluids was found to be >240°C [16]. Five months after the drilling, high-temperature hydrothermal fluid (304-311°C) discharged from the casing pipe outlet at Hole G, and the fluid discharge from the pipe continued at 25 months after drilling [17]. Prior to the drilling (2 weeks before), a thermometer and an acrylic-glass sedimentation chamber with mounting stage were placed near Hole D/E (Fig 3A). The thermometer recorded that the bottom surface temperature near Hole D/E had increased at 11 months and reached >50°C at 14–15 months after drilling, which was the maximum temperature for the thermometer (the thermometer was broken at this time, see Fig 4 in [17]).

**Fig 4:**
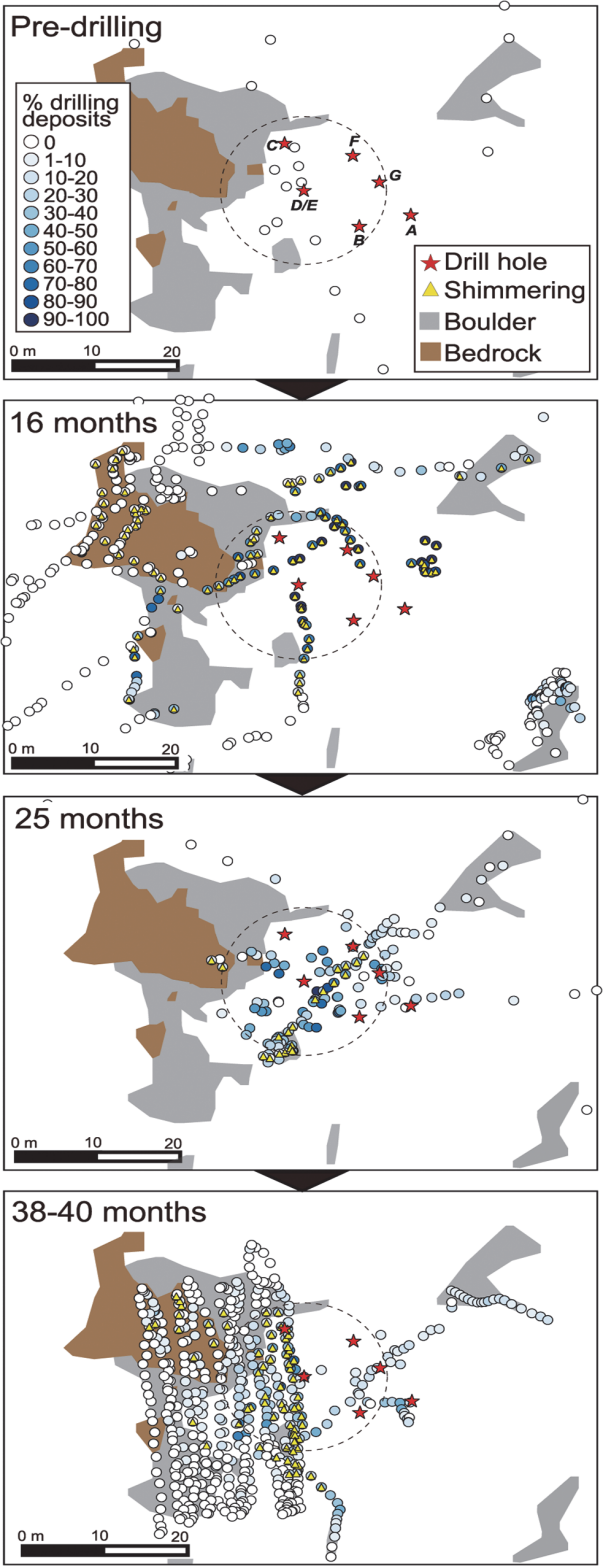
Temporal sequence of drilling deposits seen as white-color sediment as well as hydrothermal fluid discharges seen as shimmering at Site C0014. Dotted circles indicate the area within 10 m radius from Hole D/E.

### Pre- and post-drilling video observations

Pre- and post-drilling seafloor video observations were carried out 2 weeks before drilling and 11, 16, 25, 38 and 40 months after drilling using JAMSTEC’s remotely operating vehicles (ROVs) either *Hyper-Dolphin* or *Kaiko 7000 II* (Table 2). Video data were recorded with colour video cameras positioned in either vertical or oblique views by running the ROVs haphazardly around the drilling holes. At 2 weeks before and 11 months after drilling, a forward-facing video camera (Super HARP, Hamamatsu Photonic) recorded the seabed from the oblique view (the vertical distance of camera to vehicle bottom was 0.9 m). At 16, 25, 38 and 40 months after drilling, a downward-looking video camera (Handycam HDR-CX-700V, Sony) recorded the seabed vertically below the ROVs (the vertical distance of camera to vehicle bottom was 0.4 m). The surveys were conducted at an altitude of 2.8 ± 0.3 m (the average distance of vehicle bottom to the bottom substrate during the imaging for each dive) and vehicle speed of ca. 0.25 m s^-1^. The ROV angles during the video imagery were 5.1 ± 2.3° for pitch and 1.5 ± 0.6° for roll angles, thus causing some variation in subsequent measurements of seabed feature coverage and animal abundance. Positional data of ROVs from the Super-Short Baseline Navigation (SSBN) transponder were continuously recorded during the dives. The apparent outliers of the transponder were excluded before estimating the positional information of the vehicle. Position aberration of vehicles among the different survey periods were corrected based on the absolute positions of the gimballed guide base mounted on Hole G and the sedimentation chamber base placed near Hole D/E.

**Table 2:** Timing of investigations relative to the drilling event at Site C0014 in the Iheya North hydrothermal field, Okinawa Trough.

Date	Time elapsed	Cruise	ROV	Dive number
(mm/yy)				
Sep/2010	2 weeks before	NT10-E01	*Hyper-Dolphin*	1178
Sep/2010	drilling	IODP Exp331	ROV-C	
August/2011	11 months	NT11-15/16	*Hyper-Dolphin*	1312/1315
Jan/2012	16 months	KR12-02	*Kaiko 7000II*	537/538
Oct/2012	25 months	NT12-27	*Hyper-Dolphin*	1446/1447/1448/1450
Nov/2013	38 months	NT13-22	*Hyper-Dolphin*	1593/1595
Jan/2014	40 months	KY14-01	*Hyper-Dolphin*	1611

During the observation at 16 months post-drilling, in situ measurement of seawater pH was carried out using a submersible pH sensor for deep-sea (XR 420 CTD with AMP pH combined sensor, RBR Limited) which was installed on the ROV *Kaiko 7000 II*. Calibration of the pH sensor was performed pre- and post-dive operation of the ROV. In addition, hardness of the bottom sediment was examined by testing whether a push-core sampler can be inserted to the sediment (the concurrently collected sediment samples were used in other studies).

### Video data analysis

The oblique and vertical video images were used for quantitative analysis of seabed features and megabenthic animals. Video clips were captured at 10-second intervals, using the software GOM Player (Gretech) in order to provide still image frames. Overlapping and unsuitable photographs (e.g., out of focus and high sediment re-suspension) were excluded from the analysis (excluded images constituted ~ 30% of the total generated images frames).

In order to derive % coverage of disturbed sediments indicated as white-colored clay-like substrate (drilling deposits) and microbial filamentous mats seen as either white or pink, the image frame sets collected by the video cameras were imported in the image analysis software CPCe (Coral Point Count with Excel extension, [24]). For each picture frame, 50–100 random points were plotted and observed [25]. White-colored bacterial mats were impossible to distinguish from the white drilling deposits if the mat developed on or overlapped the deposits, and thus the coverage of white bacterial mats may be underestimated. The presence of fluid discharge observed as shimmering in each photograph was confirmed through cross-checking with original video clips, as it was difficult to determine if there was actually shimmering fluid from only still images. Distribution and coverage data of the discolored area and shimmering were plotted in a geographic information system using the software QGIS (version 2.2.0-Valmiera). In order to compare the seabed features (drilling deposits and microbial mats) between different sampling periods, we used the percentage cover within 10 m radius of Hole D/E as the hole was intensively visited by the ROVs.

The megabenthic invertebrate animals appeared in each image frame were identified to the lowest taxonomic levels possible and counted, and abundance of each animal was calculated from each image as number of individuals per m^2^ (inds. m^-2^). The seabed area of the image was estimated according to [26] for the perpendicular images and [27] for the oblique images using the underwater horizontal and vertical aperture angles of the camera and the camera-to-seafloor distance. We considered only animals with a minimum dimension ca. >30 mm. *Paralvinella* and polynoidea polychaetes were not counted as they were difficult to distinguish from the bottom substrate using our video observation from some distance. The distribution and abundance data of the megabenthic animals were plotted in geographical maps using QGIS. The abundances of observed animals between different sampling periods were compared within 10 m radius of Hole D/E. The statistical differences of these values were determined by multiple comparison Steel-Dwass test. Difference with *P*<0.05 was considered statistically significant.

At 16 and 40 months after drilling, carapace width length (mm) of the galatheid crab *Shinkaia crosnieri* that appeared in the perpendicular images recorded around Hole D/E was measured using the image analysis software Hakarundesu v0.7.1 (Natchan). The sedimentation chamber mount stage (base, 250 mm × 250 mm; height, 200 mm) that appeared in the images was used for length calibration. The statistical difference in the size of *S*. *crosnieri* between the different sampling periods was determined by two-tailed Student’s *t*-test. Difference with *P*<0.05 was considered statistically significant.

## Results

### Seabed landscapes before and after drilling

Prior to drilling, the sediment at Site C0014 was entirely dominated by fine-grained silty sediment (mud) (Figs 2A and 2B and 3A). Pre-drilling video surveys revealed that there were no vent endemic animals within a 15 m radius of the center of the drill holes. The hydrothermal fluid seepage community characterized by live *Calyptogena* clam colonies was the most prominent feature in the soft sediment (Fig 2B), though visual observation confirmed that more than 90% of the *Calyptogena* were dead shells. Rossellid sponges and laetmogonid holothrians were commonly observed around the clam colonies. The 15 m~ northwest of the drilling site was covered by hard substratum consisting of exposed bedrocks and boulders (Fig 2A). Diffusing hydrothermal fluids were discharged from the fissures of the exposed bedrocks at some 20–30 m west-northwest of the drilling site, where vent endemic animals *Shinkaia crosnieri* galatheid crabs and *Bathymodiolus* mussel beds were observed (Fig 2A and 2C). There was also a single small assemblage of *S*. *crosnieri* and *Bathymodiolus* mussels ca. 12 m northwest from Hole D/E (Fig 2A).

After drilling commenced the drilling process deposited white-colored clay-like sediments (probably originating from drill cuttings and mud with clay mineral, barite and bentonite) on the seabed in the vicinity of the holes (Fig 3B). This was visually confirmed at 11 months post-drilling. The multiple drilling operations drastically altered the seafloor landscape as the *Calyptogena* clam colonies were completely buried under the white-colored sediments. The white sediment extended 13–25 m from the center of drill holes at 16 months after drilling (Figs 3G and 4). The mean coverage (%) of drilling deposits seen as clay-like white sediments within a 10 m radius of Hole D/E was 60.4 ± 31.2% at 16 months after drilling. The coverage of drill deposits had decreased thereafter: 36.0 ± 21.5% at 25 months, and 20.3 ± 17.6% at 38–40 months post-drilling (Fig 4). Although it was difficult to accurately assess the thickness of the newly deposited sediments, dead clam shells that appeared inside of Hole D/E suggest this was at least ca. 300 mm (Fig 3I).

**Fig 5:**
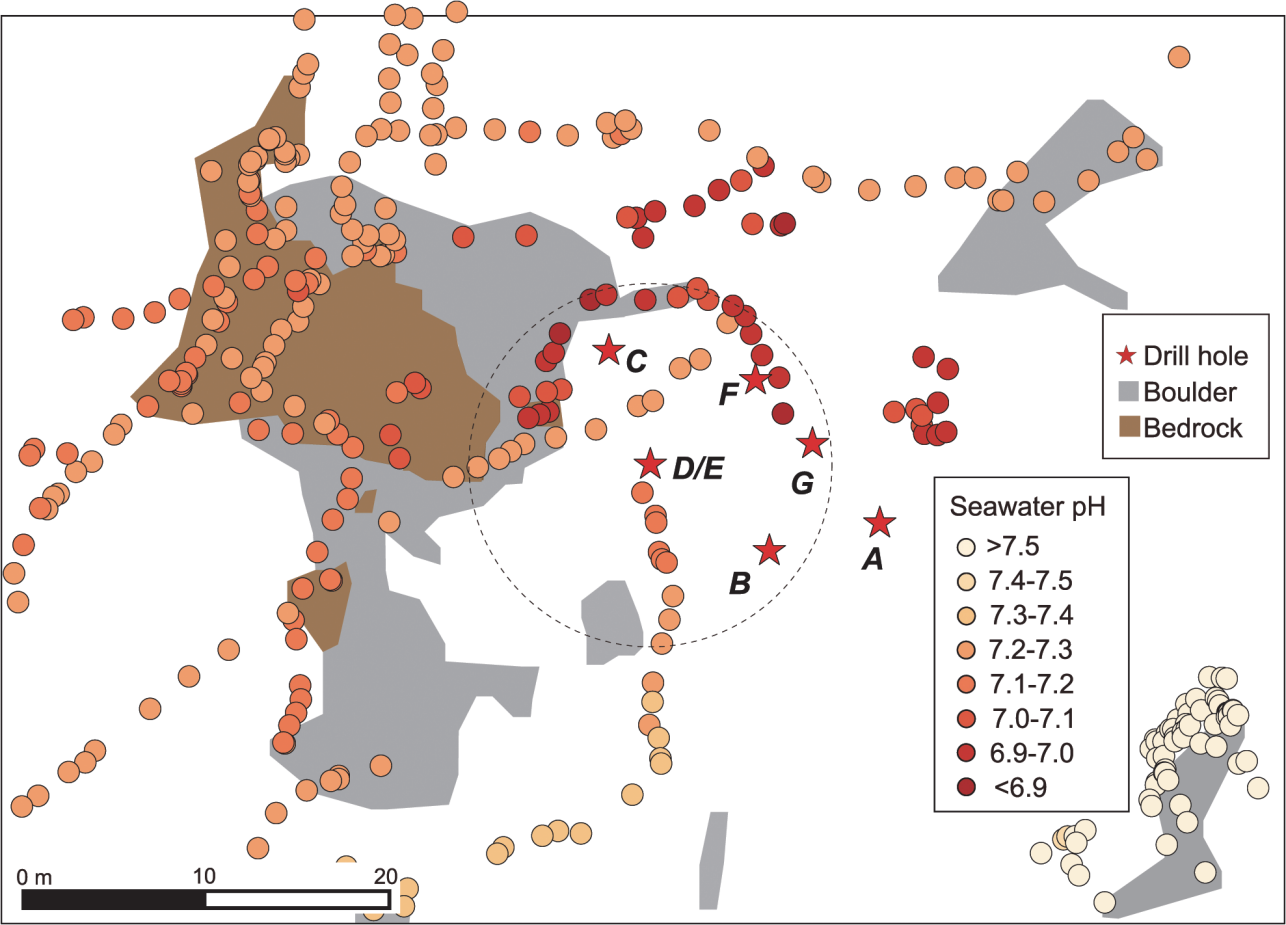
Distribution of seawater pH around drill holes at Site C0014 at 16 months post-drilling. Dotted circles indicate the area within 10 m radius from Hole D/E.

At 11 months after drilling, numerous tiny chimneys and drilling-induced hydrothermal fluid discharges seen as shimmering were observed on the white seafloor (Fig 3B, see also Fig 2L in [17]). The extent of shimmering fluid discharges induced by the drilling operations at 16 months after drilling was consistent with that of the white sediment, extending up to 24 m from the center of drill holes (Fig 4). Naturally discharged shimmering water was also recognized from exposed bedrock areas at some 20 m west-northwest the drill site at this time (Fig 4). The seawater pH showed lower values around the holes compared to the surrounding seafloor due to diffusive hydrothermal fluid discharges from the seafloor (Fig 5). At 16 months after drilling the seawater pH increased with increasing distance from the holes; 7.09 ± 0.13 within 10 m from Hole D/E, 7.12 ± 0.12 at 10–20 m, 7.22 ± 0.06 at 20–30 m, and 7.39 ± 0.17 at 30–40 m.

Upon collection of the sedimentation chamber placed near Hole D/E at 16 months, bottom of the chamber was partially melted due to the increased bottom temperature (Fig 3H). Considering the melting point of acrylic-glass [28], the seabed temperature likely have increased to at least 160°C at 16 months. At 11 and 16 months after drilling, the bottom sediment around Hole D/E was soft enough for push core-samplers to penetrate into the sediment. However, the bottom had hardened after 25 months and the area which the core-samplers could penetrate into had become limited. At 38–40 months after drilling, the bottom was further hardened and it was impossible to insert the core-sampler into the sediment around Hole D/E and the bottom substratum was visually seen to be undulating with many fissures (Fig 3F and 3L).

**Fig 6:**
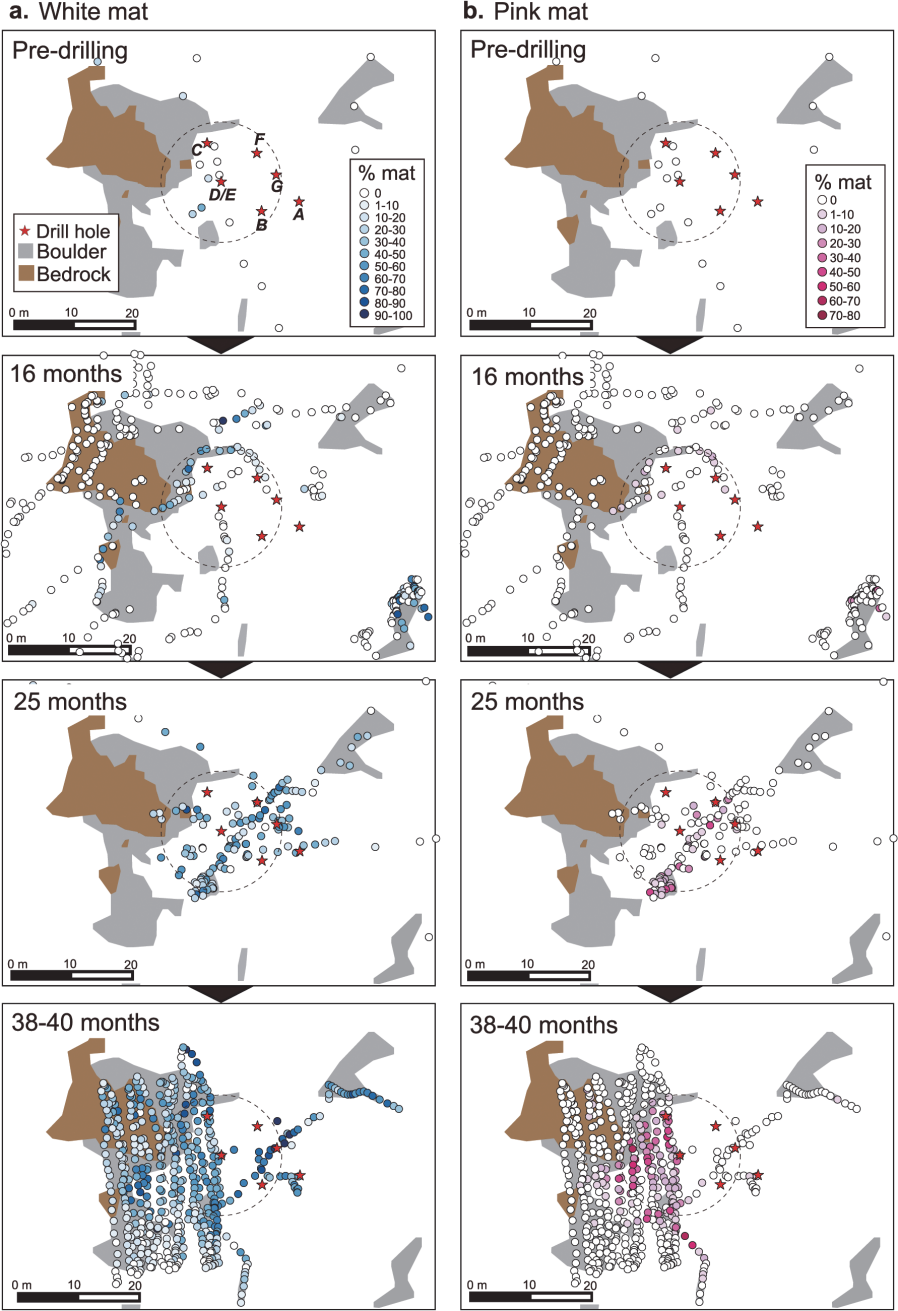
Temporal sequence of microbial mats at Site C0014. (a) white and (b) pink mats. Dotted circles indicate the area within 10 m radius from Hole D/E.

Unlike the drilling deposits, the coverage of bacterial mats around the holes increased over time (Fig 6A and 6B). Two types of microbial mats, seen as white and pink colors, have developed after drilling commenced, extending 5–25 m from the center of holes. Before the drilling commenced, white bacteria mats covered 10.3 ± 16.1% the seabed within 10 m radius from the point of Hole D/E, probably because the area hosted hydrothermal fluid seepage which also supported *Calyptogena* clam colonies. After drilling, the coverage of white bacterial mats had increased to 14.1 ± 18.4% at 16 months, 34.6 ± 19.7% at 25 months, and 46.5 ± 18.9% at 38–40 months (Fig 6A). The pink-colored bacterial mats were not observed before drilling, but were first confirmed at 16 months post-drilling, covering 1.9 ± 3.6% within 10 m radius from Hole D/E, increasing thereafter: 7.7 ± 12.5% at 25 months and 12.5 ± 16.0% at 38–40 months post-drilling (Fig 6B).

### Changes in megafaunal assemblage composition after drilling

**Fig 7:**
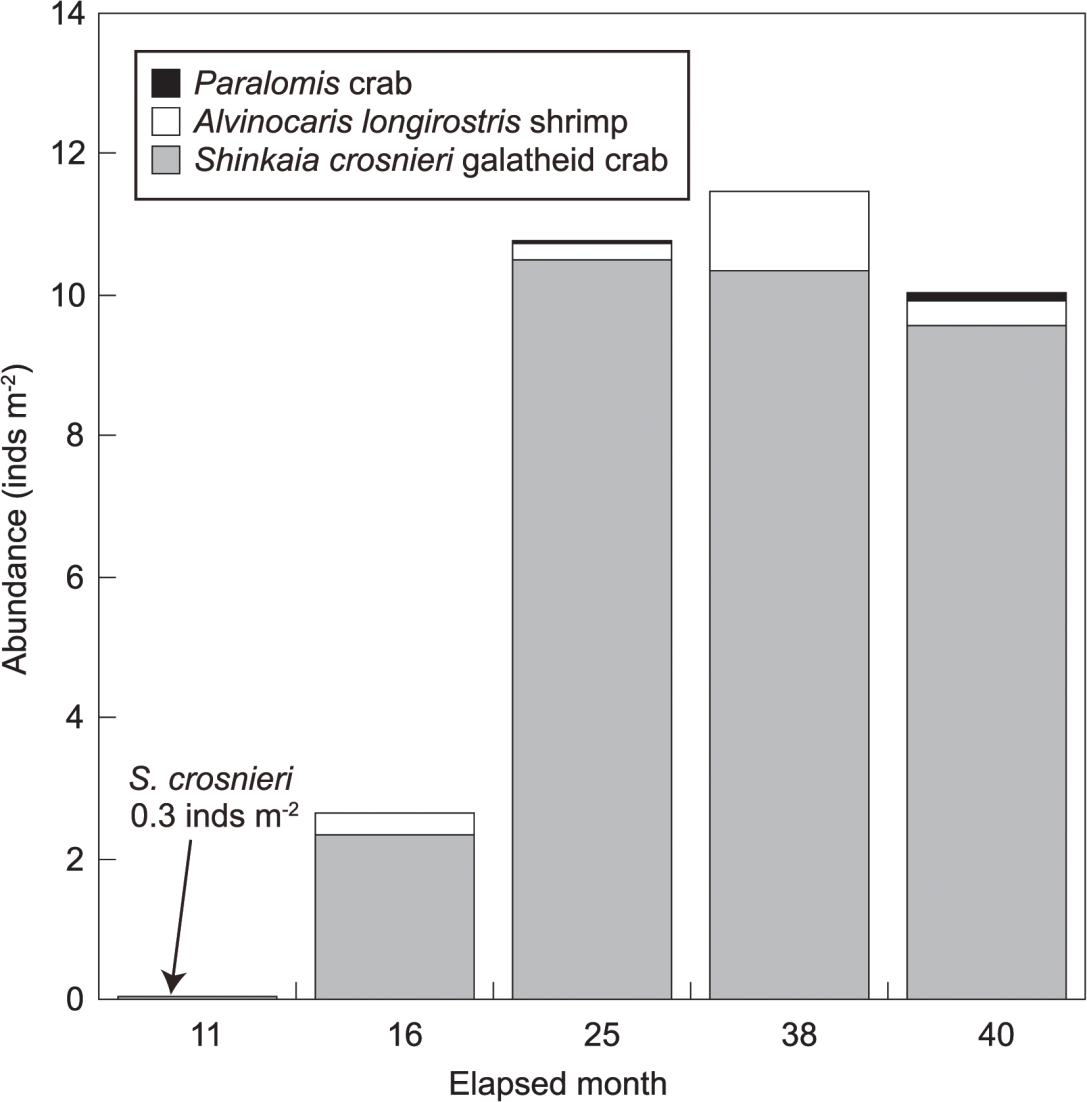
Temporal variations in the abundance of newly colonized megabenthos around Hole D/E. Abundance is the average within 10 m radius from Hole D/E.

**Fig 8:**
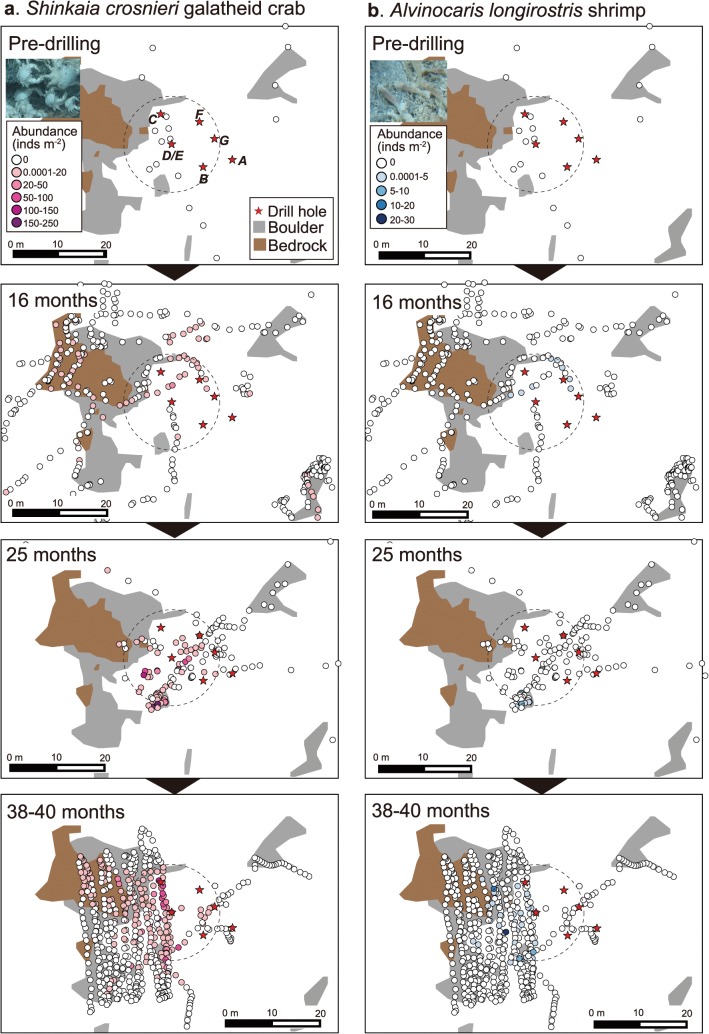
Temporal sequence in distribution and abundance of megabenthos at Site C0014. (a) *Shinkaia crosnieri* galatheid crabs and (b) *Alvinocaris longirostris* shrimps. Dotted circles indicate the area within 10 m radius from Hole D/E.

At 11 months after drilling, no benthic animals were observed 10 m radius around Hole D/E as the original *Calyptogena* clam colonies were buried under the drill deposits. At this time, however, a very low density (~0.27 inds. m^-3^) of vent endemic galatheid crab *S*. *crosnieri* was found at 7.2–9.7 m northwest of Hole D/E (Fig 7, geographical map not shown). At 16 months post-drilling the population of *S*. *crosnieri* has become widely distributed and dominated areas around the holes (Figs 7 and 8A). Another vent animal found at 16 months was *Alvinocaris longirostris* shrimp (Fig 8B). We also confirmed the presence of other vent endemic animals such as *Paralvinella* polychaetes and polynoid polychaetes at this time, although their abundance was not counted.

The main distribution of *S*. *crosnieri* was restricted within a 10–15 m radius from the holes (Fig 8A). The mean abundance of *S*. *crosnieri* within a 10 m radius of Hole D/E was 2.4 ± 7.2 inds. m^-3^ (max, 43 inds. m^-3^) at 16 months after drilling (Fig 7). Since the population of *S*. *crosnieri* was very patchily distributed, standard deviation (SD) was higher than the average. Although there were no significant differences among the different post-drilling periods (Steel-Dwass test; all test statistics, <0.6; 5% reference point, 2.57) probably due to the patchy distribution, mean abundance of *S*. *crosnieri* had increased by 4.5-fold at 25 months (mean, 10.5 ± 28.7 inds. m^-3^; max, 110 inds. m^-3^) compared to those at 16 months after drilling and became relatively stable thereafter at 38 months (mean, 10.4 ± 20.4 inds. m^-3^; max, 109 inds. m^-3^) and 40 months (9.6 ± 20.1 inds. m^-3^; max, 114 inds. m^-3^). *S*. *crosnieri* was not found inside Hole D/E at 16 months but were found inside the holes at 25 months post-drilling and thereafter (Fig 3I–3K).

**Fig 9:**
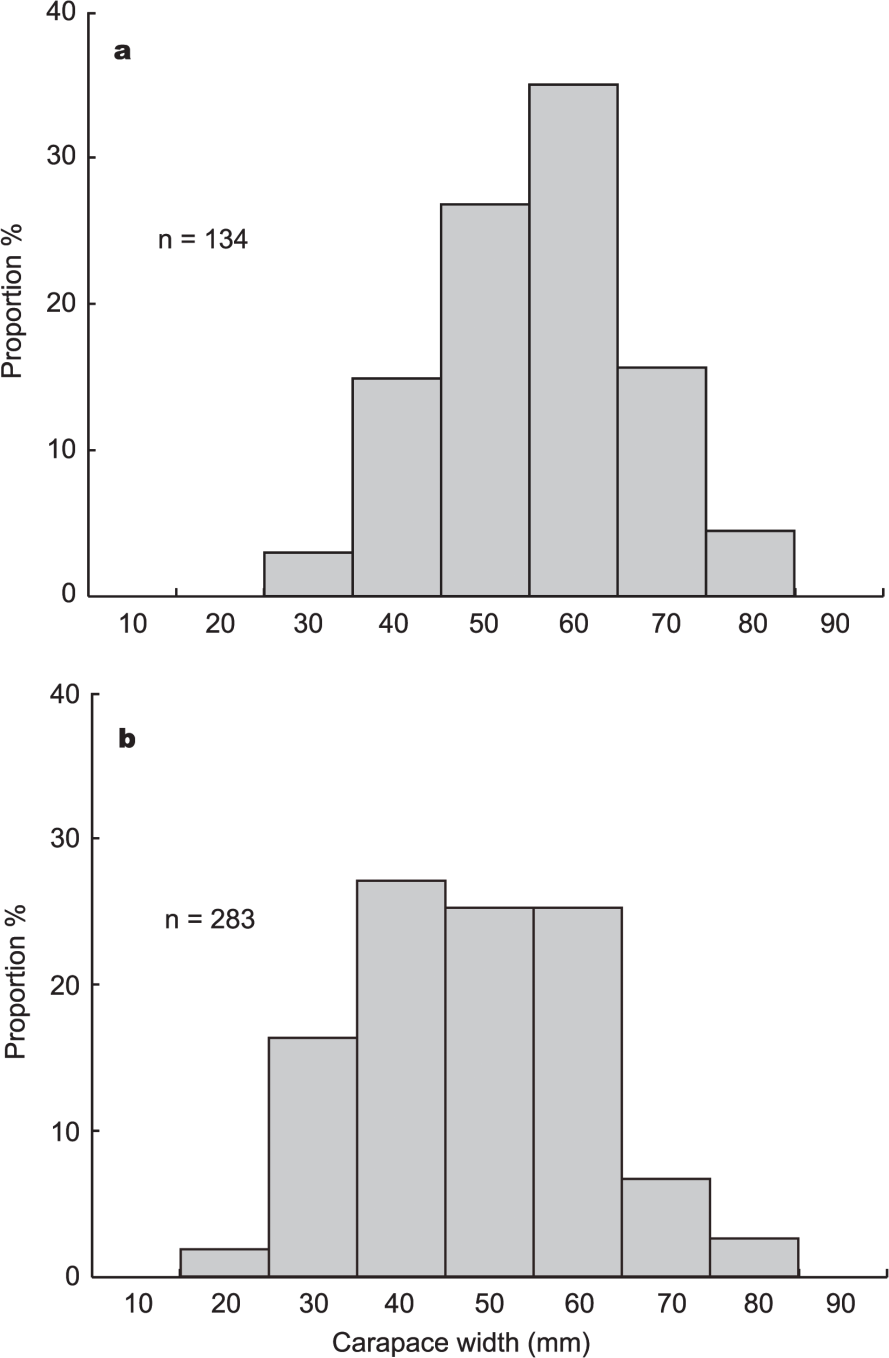
Size-frequency distribution of *Shinkaia crosnieri* galatheid crabs at (a) 16 months and (b) 40 months post-drilling. Each size-structure is based on galatheid crabs taken near Hole D/E.

The mean carapace width length of *S*. *crosnieri* at 16 months (51 ± 11 mm) was significantly (*t*-test, *P* = 0.0015×10^−6^) higher than those at 40 months after drilling (43 ± 13 mm) (Fig 9). At 16 months after drilling, larger individuals (60–70 mm width) dominated (35.1%), while at 40 months post-drilling smaller individuals (40–50 mm width) were the most predominant (25.8%) (Fig 9). The proportion of individuals with 30–40 mm carapace width was only 3.0% at 16 months, but had increased to 15.6% at 40 months. There were no individuals with 20–30 mm carapace width at 16 months, but these appeared at 40 months (1.8%).

**Fig 10:**
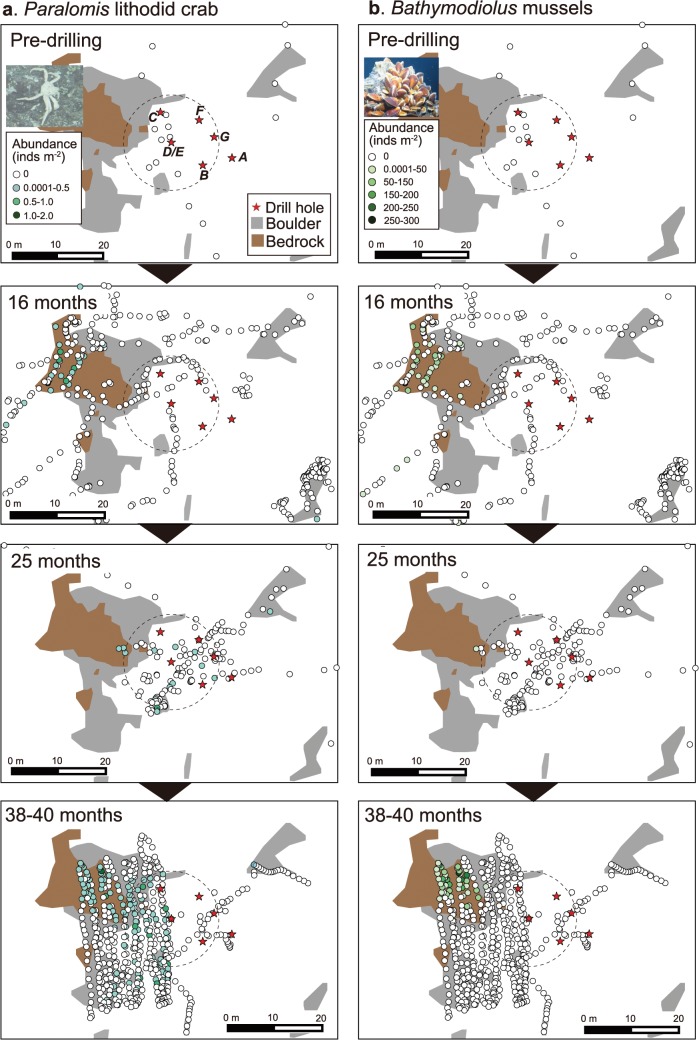
Temporal sequence in distribution and abundance of megabenthos at Site C0014. (a) *Paralomis* lithodid crabs and (b) *Bathymodiolus* mussels. Dotted circles indicate the area within 10 m radius from Hole D/E.

The mean abundance of *A*. *longirostris* shrimps at 16 months was 0.3 ± 0.8 inds. m^-3^ within 10 m radius of Hole D/E (max: 3.7 inds. m^-3^). The abundance of *A*. *longirostris* at 25, 38 and 40 months after drilling was 0.2 ± 1.2 inds. m^-3^, 1.1 ± 4.0 inds. m^-3^ and 0.4 ± 1.2 inds. m^-3^ with maximum abundance of 6.0, 21.6 and 10.0 inds. m^-3^, respectively (Figs 7 and 8B). *Paralomis* sp. was not consistently observed within a 10 m radius of Hole D/E at 16 months, but appeared at 25, 38 and 40 months after drilling (Figs 7 and 10A), with a mean abundance of 0.07 ± 0.4 inds. m^-3^ at 25 months (max: 1.0 inds. m^-3^), 0.004 ± 0.02 inds. m^-3^ at 38 months (max: 0.1 inds. m^-3^) and 0.09 ± 0.2 inds. m^-3^ at 40 months (max: 0.7 inds. m^-3^). However *Bathymodiolus* mussel, one of the typical sessile vent animals of the Okinawa Trough vents, has never been observed around the holes from our video investigations up to the most recent observation (i.e. 40 months post-drilling) (Fig 10B).

## Discussion

The results of this study revealed significant changes in the seabed landscape and megafaunal benthic community after an intensive drilling event in a deep-sea hydrothermal field. Although benthic animals that occupied the drilling site prior to drilling (such as live *Calyptogena* clams in this case) were buried under drilling deposits as reported by previous studies (e.g., [17]), the amount of megabenthic animals lost in this drilling event was much greater than those at a normal, non-chemosynthetic deep-sea floor. This is due to deep-sea chemosynthetic habitats harboring a remarkably high abundance of benthic animals (e.g., 70–1,000 ind. m^-2^, [29]) with a biomass of up to 500–1000 times greater [30,31], compared to the surrounding non-chemosynthetic sea floor (e.g., 0.2–6 inds. m^-2^, [13]).

### The extent of drilling impact on seabed landscape

The horizontal extent of the white drilling deposits in the present study was within 25 m of the drilling. This is similar to that reported from Orinoco Fan, Venezuela (10–25 m, [11]), while some other studies reported physical influence of exploitation drilling extended to approximately 100 m [9,12,13]. These differences may be due to the difference in the depths of drill holes and periods of time analyzed. Unlike any other reports though, the drilling event in the present study has subsequently caused hydrothermal fluid discharges seen as shimmering and lowering of seawater pH from the holes and the surrounding sediments, because Hole G penetrated into the subseafloor hydrothermal fluid reservoir [16]. The multiple drilling operations that disturbed the subseafloor hydrogeological structures may provide numerous vertical and lateral hydrothermal fluid pathway networks in the shallower sediments surrounding the holes [17].

During the study period, deposits from the drilling event have been gradually removed probably by lateral advection during high current events and from vertical redistribution in the sediments [12]. In contrast, bacterial mats have developed and become widespread. The newly emerged hydrothermal fluid discharges increased hydrothermal fluid inputs such as methane and hydrogen sulfide and increased the temperature of seafloor surface, promoting bacterial mat growth [32]. An increase in the areal extent of microbial mats up until 40 months post-drilling indicates a continuous supply of hydrothermal fluids from the subseafloor sources. This phenomenon differs from the microbial mat succession observed at the East Pacific Rise volcanic eruption in 1991, which reported a marked reduction in the areal coverage of the microbial mats within one year (from >20 m^2^ to <5 m^2^) and no longer present 32 months after the eruption due to the reduction of hydrothermal flux [7]. Considering the horizontal extent of the shimmering fluid and white drilling deposits as well as newly developed bacterial mats, the horizontal impact distance by the scientific drilling was probably less than 30 m.

Seabed around the drill holes has been hardening throughout the post-drilling period, becoming rough and undulated with many fissures at 38–40 months after drilling. Unfortunately, we did not have sediment samples to characterize the hardening process in this study. However, the most likely process is barite/gypsum precipitation or silicification of the seafloor sediment. As mentioned earlier, high-temperature hydrothermal fluid (>160°C) discharged through the seafloor sediment. If this hot fluid was mixed with ambient cold (4°C) seawater, sulfate minerals such as anhydrite (CaSO_4_), gypsum (CaSO_4_•2H_2_O) and barite (BaSO_4_) could be precipitated. The solubility of anhydrite in the ocean has a reverse relationship as a function of the temperature [33]. Thus, most of the anhydrite would be dissolved in the water when the seabed temperature decreased to ambient seawater temperature as previously observed at another IODP 331 drilling site (Site C0013, [17]), while some may remain as gypsum. Conversely, the solubility of barite is approximately three orders of magnitude lower than that of anhydrite [34], leading to survival of barite crystals. Indeed, a barite-rich chimney sample has been collected at the Iheya North field in the past, prior to the present study [35]. Barite also originated from drilling mud, which is used together with bentonite to adjust the density of circulative drilling mud water. Therefore, some fractions of barite crystal that harden the seabed may be of an artificial origin. Such barite/gypsum precipitation or silicification of the seafloor sediment might have sealed and plugged the hydrothermal paths within the sediment, which in turn converged the subseafloor hydrothermal flow. This process is a possible factor for the numerous fissures observed at 38–40 months, due to fluid pressure. Since bentonite is well known to produce low permeable layer that shut out the flow [36–38], artificially added bentonite in the drilling mud fluid may have also contributed to converge the subseafloor hydrothermal flow. Further investigation and additional sediment sampling are necessary to elucidate the detailed mechanism of the seabed hardening after drilling.

### Megafaunal assemblages at the artificial hydrothermal vents

Previous studies on the deep-sea drilling impacts in oil and gas fields showed that megafaunal density and diversity recovers partially from drilling disturbance after 3 years as there was significant removal of cuttings from those initially deposited [12]. Contrariwise, the disturbed ecosystems in the present study have not recovered to the pre-disturbed condition after 3 years, due to newly emerged hydrothermal fluid discharges from the subseafloor establishing new hydrothermal vent ecosystems. The ‘artificial’ vent ecosystem created has already lasted 2 years and is likely to be maintained as long as the hydrothermal fluid supply continues.

Originally, the drilling site was characterized by a chemosynthetic community sustained by hydrothermal fluid seepage (geofluid seep), where *Calyptogena* clams dominated. The clam colonies were however completely buried under the drilling deposits, and converted to a community consisting of typical vent-associated animals after several months. The galatheid crab *S*. *crosnieri* was predominant in the ‘artificial’ vent community, and their abundance around Hole D/E increased considerably from 11 months to 25 months, stablizing thereafter. We confirmed that a small number of *S*. *crosnieri* at 11 months within 10 m of Hole D/E and subsequent higher abundance at 16 months after drilling. This suggests the galatheid crabs started migrating to the drill site from 11 months when the bottom surface temperature around the holes started to increase, indicating hydrothermal fluid discharges [17]. Compared to the population at 40 months post-drilling, most individuals of *S*. *crosnieri* were larger in size in the 16 months post-drilling population, which also lacked small individuals (<3 cm carapace width). This strongly indicates that *S*. *crosnieri* migrated on foot to the drill site from existing nearby colonies by sensing the fluid discharge (chemistry and/or lowering of seawater pH), rather than through planktonic larvae dispersal. Although it may be possible for planktonic larvae of *S*. *crosnieri* to arrive at the drill site after 11 months, it is unlikely that individuals developed from planktonic larvae to adult in this short period (5 months, from 11 to 16 months) considering the general crustacean growth rate [39]. Previous seabed observations confirmed several colonies of *S*. *crosnieri* outside of 20–40 m radius of the drill holes, implying their ability to migrate at least 20 m distance on foot.

Similarly, another typical vent animal *Alvinocaris* shrimp was confirmed at 16 months after drilling. *Paralomis* lithodid crabs have appeared at 25 months after drilling. *Paralomis* crabs are typical carnivores [23] and may have migrated into the drilling site following the migration of their potential preys, possibly *Shinkaia* galatheid crabs. Yet, another representative vent animal in the Iheya North field, *Bathymodiolus* mussels, has never been observed around Hole D/E throughout the study periods even when there are several mussel bed colonies at ca. 12–30 m distance around the drill holes. Although the sessile *Bathymodiolus* mussels can move short distances by depositing and releasing byssal threads [40,41], it is unlikely that they move such a long distance (20~ m) on foot. Consequently they can probably only migrate to the drill site through planktonic larvae dispersal. Assuming that the larvae had a steady growth of ca. 2 cm year^-1^ [42], if they had settled after establishment of the ‘artificial’ vent community, these mussels must be visually recognizable from our ROV camera observation within 40 months after drilling. This strongly indicates that the drill site is unsuitable for mussel colonization. In the Okinawa Trough, *Shinkaia* crabs, *Alvinocaris* shrimps and *Paralvinella* polychaetes have been reported as being closely associated with active vents, while *Bathymodiolus* mussels occur distantly from active vents probably due to their lower tolerance levels of temperature and/or toxic substances such as hydrogen sulfide [43]. The high bottom temperature and/or high concentrations of hydrogen sulfide at the drill site may not provide suitable habitat for the mussels.

## Conclusions

In conclusion, the intensive drilling campaign in the Iheya North hydrothermal field resulted in the complete collapse of the original hydrothermal-fluid-seepage community visually characterized by *Calyptogena* clams (some alive) due to drilling deposits. The subsequent ‘artificial’ hydrothermal fluid discharges from the holes and the surrounding seabed created ‘artificial’ hydrothermal vent ecosystems. The vent-endemic galatheid crab *S*. *crosnieri* quickly migrated to the newly created habitats and dominated the new vent community. Bottom substrate had hardened probably due to barite/gypsum mineralization or silicification, becoming rough and undulated with many fissures after drilling operation. Although the impacts of the drilling operation on seabed landscape and megafaual invertebrate composition were confined to an area of 30 m maximum from the drill holes, the newly established ‘artificial’ vent ecosystem from these drillings is likely to continue to exist until the fluid discharge ceases. Consequently, ecosystem in this area has been altered for long-term. It is indefinite how violent the disturbances to seabed will be, in the case of full-scale commercial mining. Such mining and their prerequisite feasibility studies of SMS deposits may require a large number of subseafloor drilling, for mineral deposit reserve assessment. Therefore, massive commercial mining anticipated in the near future will potentially create more ‘artificial’ hydrothermal vent ecosystems at a much greater scale.
